# Role of Microglia TLRs in Neurodegeneration

**DOI:** 10.3389/fncel.2018.00329

**Published:** 2018-10-02

**Authors:** Bernd L. Fiebich, Carla Ribeiro Alvares Batista, Soraya Wilke Saliba, Nizar M. Yousif, Antonio Carlos Pinheiro de Oliveira

**Affiliations:** ^1^Neuroimmunology and Neurochemistry Research Group, Department of Psychiatry and Psychotherapy, Medical Center—University of Freiburg, Faculty of Medicine, University of Freiburg, Freiburg, Germany; ^2^Department of Pharmacology, Universidade Federal de Minas Gerais, Belo Horizonte, Brazil; ^3^Faculty of Biology, University of Freiburg, Freiburg, Germany

**Keywords:** toll-like receptors (TLRs), microglia, neurodegenerative diseases, neuroinflammation, Alzheimer’s disease, Parkinson’s disease, inflammatory mediators

## Abstract

Toll-like receptors (TLRs) are a group of receptors widely distributed in the organism. In the central nervous system, they are expressed in neurons, astrocytes and microglia. Although their involvement in immunity is notorious, different articles have demonstrated their roles in physiological and pathological conditions, including neurodegeneration. There is increasing evidence of an involvement of TLRs, especially TLR2, 4 and 9 in neurodegenerative diseases such as Alzheimer’s disease (AD), Parkinson’s disease (PD), and amyotrophic lateral sclerosis (ALS). In this sense, their expression in microglia might modulate the activity of these cells, which in turn, lead to protective or deleterious effects over neurons and other cells. Therefore, TLRs might mediate the link between inflammation and neurodegenerative diseases. However, further studies have to be performed to elucidate the role of the other TLRs in these diseases and to further prove and confirm the pathophysiological role of all TLRs in neurodegeneration. In this article, we revise and summarize the current knowledge regarding the role of TLRs in neurodegeneration with the focus on the possible functions of these receptors in microglia.

## General Aspects of Toll-Like Receptors

Toll-like receptors (TLRs) are a growing family that plays critical roles in innate immune responses. Several TLRs have already been identified in human and murine, such as TLR1–5 (Chaudhary et al., 1998; Rock et al., 1998), TLR6 (Takeuchi et al., 1999), TLR7–9 (Du et al., 2000). TLRs are located on the plasma membrane with the exception of TLR3, TLR7, TLR9 that are presented intracellularly in vesicles such as the endoplasmic reticulum, endosomes or lysosomes (Kawai and Akira, 2010).

## Signaling

TLRs are activated by different pathogen-associated molecular patterns (PAMPs), including compounds derived from bacteria, virus, or fungi (Takeuchi et al., 2002; Li et al., 2013). TLR signaling pathways depend on receptor dimerization and recruitment of adapter proteins that mediate other protein-protein interactions, such as myeloid differentiation primary-response protein 88 (MyD88) and Toll/interleukin 1 receptor (TIR) domain-containing adaptor interferon-β (TRIF; Takeda et al., 2003; Yamamoto et al., 2003a). Importantly, all TLR signaling pathways are dependent on MyD88, except TLR3, which initiates signaling via the TRIF adapter. In addition, TLR4 is activated by both MyD88-associated and TRIF-related adapter molecule (TRAM)-associated pathways (Fitzgerald et al., 2003a; Ruckdeschel et al., 2004).

The MyD88-dependent pathway initiates with the recruitment of tumor-necrosis-factor-receptor-associated factor 6 (TRAF6) and members of the IL-1R-associated kinases (IRAK) family. The activation of TRAF6 allows the translocation of nuclear factor kappa B (NF-κB) to the nucleus, which results in the production of different pro-inflammatory mediators such as cytokines and cyclooxygenase-2 (COX-2; Zhang et al., 1999; Takeda and Akira, 2004; Broad et al., 2007; Kawai and Akira, 2010).

While the MyD88-dependent pathway promotes the production of pro-inflammatory cytokines, the MyD88-independent pathway is associated with the induction of interferon (IFN)-inducible genes and maturation of dendritic cells (Yamamoto et al., 2003b). The MyD88-independent pathway is mediated by TRAM and TRIF. Inhibitor of NF-κB kinase subunit epsilon (IKKε) and TANK-binding kinase (TBK) 1 interact with TRIF or TRAM and activate interferon regulatory factor 3 (IRF-3) and NF-κB-dependent signaling pathways (Fitzgerald et al., 2003b; Palsson-McDermott and O’Neill, 2004). IRF3 mediates the expression of IFN type I, e.g., IFN-β and chemokines, such as C-C motif chemokine ligand 5 (CCL5) and C-X-C motif chemokine ligand 10 (CXCL10; Schafer et al., 1998; Melchjorsen and Paludan, 2003).

## TLR Expression in the CNS

TLRs are widely expressed in the CNS and play different roles in cell survival or death (Okun et al., 2011). Studies have demonstrated that TLRs are expressed in neurons (Tang et al., 2007), microglia (Olson and Miller, 2004), astrocytes (Bowman et al., 2003), oligodendrocytes (Lehnardt et al., 2006) and neural stem cells (NSC; Okun et al., 2009; van Noort and Bsibsi, 2009). It has been demonstrated that NSC transplantation in APP/PS1 mice decreased inflammatory injury through suppression of glial and TLR4-mediated inflammatory pathway activation (Zhang et al., 2016). The expression of TLRs is affected by aging, which is a risk factor of neurodegenerative diseases, such as Alzheimer’s disease (AD). Aged cultures of rat hippocampal neurons presented an increase of TLR4 expression (Calvo-Rodríguez et al., 2017) while the expression levels of TLR2 and TLR4 increased during the suckling period and then decreased once the infants reached 1 year of age (Savino et al., 2018).

Microglia are the resident mononuclear immune cells of the CNS and play important roles in the maintenance of homeostasis and neuroinflammation (Ransohoff and Perry, 2009; Perry et al., 2010; Boche et al., 2013). Microglia express all TLRs and their adapter proteins as shown in mice, rats, and humans (Bsibsi et al., 2002; Olson and Miller, 2004; Zhang et al., 2013), such as TLR1 and TLR6 (Bsibsi et al., 2002), TLR2 (Laflamme et al., 2001), TLR3, TLR5, TLR7, TLR8 (Bsibsi et al., 2002), TLR4 (Laflamme and Rivest, 2001) and TLR9 (Dalpke et al., 2002). Constitutive expression of TLRs is primarily in microglia and largely restricted to the circumventricular organs (CVOs) and meninges, areas with direct access to the circulation, although they may also be expressed at lower levels in other regions (Laflamme et al., 2001; Laflamme and Rivest, 2001; Chakravarty and Herkenham, 2005).

Different stimuli may lead to increased expression of microglia TLRs, such as hypoxia (Smith et al., 2013; Yao et al., 2013), LPS (Kielian et al., 2005; Yousif et al., 2018), Poly(I:C; Yousif et al., 2018), kainic acid (Wang et al., 2015; Hu and Mao, 2016), α-synuclein (Béraud et al., 2011; Daniele et al., 2015) and amyloid beta (Aβ; Jana et al., 2008; Richard et al., 2008; Caldeira et al., 2017).

## TLRs in Neurodegeneration

TLR activation can be beneficial or detrimental to the host. In addition to the known role of being pro-inflammatory in the protective response against pathogens in the CNS, TLRs may be activated in the absence of microbial invaders. It has been shown that TLRs are upregulated in brains of AD patients (Liu et al., 2005) and other neurodegenerative diseases (Bsibsi et al., 2002; Walter et al., 2006), such as amyotrophic lateral sclerosis (ALS) and Parkinson’s disease (PD; van Noort and Bsibsi, 2009; De Paola et al., 2012; Lee et al., 2015). An increased expression of TLR2, TLR4, TLR5, TLR7, TLR9 and the TLR co-receptor CD14 has been shown in microglia cells situated around senile plaques in post-mortem brain of AD patients and related mouse models (Liu et al., 2005; Walter et al., 2007; Jana et al., 2008; Letiembre et al., 2009). Another study demonstrated a significant alteration of cognitive functions in TLR2-deficient APP/PS1 mice and synaptic plasticity associated with increased levels of toxic Aβ1–42 (Richard et al., 2008). Further studies associated TLRs with ALS in mouse models, suggesting their crucial role in the neurodegenerative process of this pathological condition (De Paola et al., 2012; Lee et al., 2015).

Finally, a crosstalk between TLR and cell stressors has been observed in immune cells, such as the unfolded protein response (UPR) and oxidative stress, which are present in different neurodegenerative diseases (Qin et al., 2005; Gill et al., 2010; Gough, 2010; Coope et al., 2012; Grootjans et al., 2016; Sprenkle et al., 2017; Wang et al., 2017). Besides, various components present in neurodegenerative diseases, which will be discussed below, activate the TLRs present in microglia. In this sense, many stimuli modulate the activity of these receptors and alter microglia phenotype and activation. Thus, TLRs present in microglia may partially mediate the development of these pathological conditions.

## TLR2

TLR2 is one of the most studied TLR in respect to neurodegenerative diseases (Guillot-Sestier and Town, 2018). In microglia, the coupling of CD14 to TLR2 and TLR4 is necessary for the induction of immune response associated with fibrillar Aβ phagocytosis (Reed-Geaghan et al., 2009). Moreover, it was reported that activation of TLR2 in microglia enhances pathological Aβ uptake (Chen et al., 2006). However, others showed that inhibition of TLR2 activation decreased glial cell reactivity, leading to reduced Aβ deposits and improved cognitive performance in APP/PS1 mice (McDonald et al., 2016). Likewise, knocking out TLR2 in mice prevented memory impairment occurring after Aβ1–42 immunization (Vollmar et al., 2010). This is supported by results from cultured microglia showing that TLR2 deficiency reduced Aβ42-induced inflammation and enhanced Aβ clearance (Jana et al., 2008; Liu et al., 2012). In accordance, TLR2-dependent JNK/NF-κB signaling partially contributed to the inflammatory response mediated by Aβ1–42 (Lin et al., 2013). Immunohistochemistry data demonstrated the TLR2 localization on microglia associated with Aβ plaques in old APP23 transgenic mice (Frank et al., 2009). Genetic studies identified TLR2 as one of the risk factors associated with Late-Onset AD (LOAD) in Azeri Turk ancestry and Han Chinese populations (Wang et al., 2011; Yu et al., 2011a; Rezazadeh et al., 2016). However, results from another report argue against the relationship between TLR2 polymorphism and LOAD (Yu et al., 2011b).

The potential role of TLR2 in PD is supported by the existing link between TLR2 polymorphism and increased risk of PD (Kalinderi et al., 2013). α-synuclein released by neurons extracellularly acts as an endogenous ligand for TLR2 on microglia, which in turn produce toxic molecules leading to neurodegeneration (Kim et al., 2013, 2016). In mice, overexpression of α-synuclein increased TLR2 expression and led to increased microglial activation (Drouin-Ouellet et al., 2015). Disease stage and region-dependent expression of TLR2 consistent with region-specific phenotype and activation of microglia shown in different groups of PD patients suggest a critical role of TLR2 in the microglia-related responses observed in PD (Doorn et al., 2014). Upon treatment with α-synuclein, microglia exhibit pro-inflammatory phenotype by forming a heterodimer complex that engage TLR1 and TLR2, leading to a chronic neuroinflammatory milieu. In this context, candesartan cilexetil, a drug used for treatment of hypertension also known to suppress the expression of TLR2, reversed the pro-inflammatory microglia phenotype activated by α-synuclein exposure (Daniele et al., 2015). Interestingly, TLR2 and its downstream signaling were shown to play a key role in the neuroprotective effects achieved through exercise approaches in 1-methyl-4-phenyl-1,2,3,6-tetrahydropyridine (MPTP) mouse model of PD (Jang et al., 2017; Koo et al., 2017).

Furthermore, induced expression of TLR2 was detected strictly within microglia in the chronically LPS-stimulated SOD1 transgenic mouse model of ALS. The increased TLR2 expression was strongly correlated with robust immune response and degeneration of motor neurons and motor axons (Nguyen et al., 2004). Another report demonstrated that overexpression of ALS-related SOD1 mutant promotes microglia neurotoxic effects via TLR2 (Liu et al., 2009). The fact that marked upregulation of TLR2 has already been observed in a previous study implemented in the ALS mouse model is in line with these findings (Nguyen et al., 2001). In two culture models of mSOD1-linked ALS, microglia TLR2 dependent activation was required to induce toxic effects and injury of motoneurons (Zhao et al., 2010). Increased expression of TLR2 in reactive glial cells was detected in spinal cord of ALS patients. Interestingly, TLR2 was mainly detected in myeloid cells (microglia, macrophages; Casula et al., 2011). Taken together, the role of microglial TLR2 in ALS seems to be consistent, since data from various reports are in agreement. This fact also suggests that modulation of TLR2 in ALS might be a potential target for effective therapy.

## TLR3

TLR3 is less studied in respect to neurodegenerative diseases, although it may be involved in neuroinflammation. For example, incubation of microglia with Poly(I:C), a TLR3 agonist, increases the synthesis of COX-2, mPGES-1 and prostaglandin E_2_ release (de Oliveira et al., 2016), and increased the expression of TLR3 (Yousif et al., 2018). The production of these inflammatory mediators might contribute to neurodegenerative processes observed in some conditions. Indeed, a previous study shows that peripheral challenge with Poly(I:C) increased Aβ levels in the hippocampus linked to cognitive dysfunction in non-transgenic mice (Weintraub et al., 2014). Moreover, systemic challenge with Poly(I:C) amplified CNS inflammation and exacerbated chronic neurodegeneration in the ME7 model of prion disease (Field et al., 2010). Importantly, a very recent study in human AD brains identified increased expression of TLR3 in microglia associated with Aβ plaques (Walker et al., 2018). In addition, TLR3 is an essential receptor for viral detection (Hanke and Kielian, 2011). It has already been shown that influenza virus infection may lead to neurodegeneration (Jang et al., 2009). Other viruses such as hepatitis C virus, Eppstein-Barr virus and human immunodeficiency virus (HIV) have been also linked to PD (Woulfe et al., 2014; Moulignier et al., 2015; Wu et al., 2015).

## TLR4

An elegant and recent study demonstrated that microglia immune memory shapes neuropathology and provided potential mechanism for LPS-triggered immune memory in the brain, suggesting a role of TLR4 in this phenomenon (Wendeln et al., 2018). Interestingly, both, beneficial and detrimental roles of TLR4 in the context of AD are reported. Transgenic AD mice deficient in TLR4 exhibit reduced microglial activation, increased Aβ deposition, and reduced cognitive function (Song et al., 2011). In the transgenic mouse model of tau pathology P301S, chronic mild stimulation of TLR4 with LPS reduced cerebral p-tau proteins and improved memory impairment through microglia-dependent activation of autophagy (Qin et al., 2016). In contrast, activation of TLR4 but not TLR2 reduced microglial Aβ42-clearance through modulation of the scavenger receptor CD36 activity (Li et al., 2015). Pronounced TLR4 expression in APP mice and an increased expression of TLR4 in brains of AD patients associated with Aβ plaques have been identified (Walter et al., 2007). TLR4 was shown to be necessary for glial cell activation that resulted in impairment in memory in a mouse model of AD based on acute Aβ injection (Balducci et al., 2017). A recent report suggests that alteration in microglial TLR4 signaling contribute to AD pathogenesis in APP/PS1 mice (Go et al., 2016).

Collectively, the role of TLR4, likewise TLR2, in AD is perhaps disease-stage dependent and this could be at least one explanation for the controversy found in the literature about the impact of these receptors in AD pathology. In this regard, activation of TLR4 in the initial stage has beneficial effects on Aβ-clearance, while chronic activation contributes to Aβ aggregation (Huang et al., 2017).

Previous studies suggest that TLR4 deregulation may play an important role in α-synucleinopathies (Caputi and Giron, 2018). TLR4 was shown to be essential for microglia to induce responses against α-synuclein *in vitro* such as uptake of this protein, pro-inflammatory cytokine release, and reactive oxygen species production (ROS; Fellner et al., 2013). TLR4 deficiency in the MPTP induced PD model resulted in reduced cell death and decreased number of reactive microglia (Noelker et al., 2013). Impaired microglial phagocytosis, enhanced neuronal loss and exacerbated motor dysfunction upon TLR4 ablation has been shown in a transgenic mouse model of multiple system atrophy with oligodendroglial α-synuclein overexpression (Stefanova et al., 2011). Moreover, stimulation of TLR4 with monophosphoryl lipid A in mice overexpressing α-synuclein increased microglia phagocytosis of this protein, improved motor deficits, and prevented neuronal degeneration (Venezia et al., 2017). In BV2 microglia-like cells, TLR4-dependent immune response regulated Nurr1 and NF-κB signals, which were shown to be vital during neuroinflammation stimulated by α-synuclein (Shao et al., 2018). In addition, TLR4 signaling was shown to exert a crucial function in the activation of BV2 cells and the promotion of inflammation induced by 1-methyl-4-phenylpyridinium (MPP(+); Zhou et al., 2016). In an* in vitro* model of PD, neuron-derived IgG mediates dopaminergic neurons survival by reducing apoptosis induced by 6-hydroxydopamine toxicity through a mechanism that involved the activation of microglial TLR4 (Zhang et al., 2013). Taken together, TLR4 seems to exert different roles in relation to PD. Nevertheless, future investigations aiming to target TLR4 in a specific cell subset in the brain will provide a better understanding concerning the role of this receptor in the context of PD.

Recently, the impact of TLRs in respect to ALS has been given some attention. For instance, in the hSOD1 G93A mouse model of ALS, increased TLR4 expression has been detected in microglia and astrocytes. In the same model, TLR4 deficiency improved motor function and extended life expectancy (Lee et al., 2015). *In vitro* and *in vivo* approaches of motor neuron degeneration, treatment with TLR4 antagonist decreased microglial activation, exerted neuroprotective effects, and improved behavior performance (De Paola et al., 2012). In line with these findings, antagonizing TLR4 in motoneurons/glia co-cultures isolated from SOD1 G93A mice prevented cell death induced by SOD1mut glia (De Paola et al., 2016).

The role of TLRs in Huntington’s disease (HD) is not covered. There is only one study that directly addressed the importance of TLR2, TLR3 and TLR4 in the N171–82Q mouse model of HD. In this study, when TLR2, TLR3 and TLR4 were lacking, the survival rate of HD mice was increased (Griffioen et al., 2018).

## TLR5

In the context of neurodegenerative diseases, Bsibsi et al. (2002) have demonstrated that human microglial cell cultures from donors with AD, olivopontocerebellar atrophy (OPCA), and Pick’s disease (PID) expressed increasing levels of TLR5 but differing between the donors. Additionally, overexpression of TLR5 in the substantia nigra, striatum, cerebral cortex and nucleus dentatus areas, was observed in multiple systems atrophy patients (Brudek et al., 2013) and in the brain stem of a genetic PD mouse model (Letiembre et al., 2009). In contrast, down-regulation of TLR5 expression was observed in the frontal cortex of a PD rat model, using intranasal administration of MPTP (Viana et al., 2017). However, additional studies are needed to elucidate the effects of TLR5 in neurodegenerative diseases and to comprehend its role in microglia.

## TLR7

The expression of TLR7 has been demonstrated in different neurodegenerative diseases (AD, PD, OPCA and PID) and in disease models including human microglial cell culture (Bsibsi et al., 2002) or murine microglial cells (Béraud et al., 2011; Béraud and Maguire-Zeiss, 2012). Furthermore, the expression of TLR7 has also been demonstrated in some areas of the CNS of genetic mice models of AD (brain cortex) and ALS (spinal cord; Letiembre et al., 2009; Liu et al., 2017). Other studies have demonstrated that TLR7 activation induced neurodegeneration. Rosenberger et al. (2014) have shown that activation of TLR7 in neurons, but not in microglia, significantly enhanced apoptosis in co-cultures and the TLR7 agonist (loxoribine) induced loss of neurons, axonal injury in the cerebral cortex and pronounced microglia activation in the mouse brain. Another study has verified the activation of TLR7 by microRNA let-7, a regulator of gene expression in the CNS, which induced neurodegeneration independent of microglia (Lehmann et al., 2012a). In contrast, the same group has shown TLR7 activation by ssRNA40-induced neuronal cell death through microglia (Lehmann et al., 2012b).

In a transgenic mouse model of AD, deletion of TLR7 ameliorates spatial learning, but did not affect microglia activation, cytokine expression, or Aβ deposition (Liu et al., 2017).

Nevertheless, the activation of TLR7 has been described to induce autophagy (Delgado et al., 2008) and has been proposed to be involved in the Aβ clearance by microglia (Gambuzza et al., 2014). Further studies have to be performed to elucidate the function of TLR7 in autophagy and its impact in neurodegenerative diseases.

At present, the role of microglial TLR7 in neurodegenerative diseases remains elusive. However, one potential strategy to address this issue would be to use a TLR7 knockdown model specifically in microglia and associate it with models of neurodegeneration.

## TLR9

TLR9 is overexpressed in brain regions such as substantia nigra and putamen from PD patients (Ros-Bernal et al., 2011), in striatum of a PD mouse model (Ros-Bernal et al., 2011), and in the spinal cord in an ALS mouse model (Letiembre et al., 2009).

Some studies have demonstrated that the activation of TLR9 signaling exacerbate neurodegeneration by inducing oxidative stress and inflammation. CpG-DNA activated microglia and induced TNF-α and nitric oxide (Dalpke et al., 2002; Ebert et al., 2005). In a co-culture containing microglia and neurons, the activation of microglial cells by CpG-DNA via TLR9 induced neuronal toxicity mediated partially through TNF-α (Iliev et al., 2004). Intracerebroventricular infusions of CpG-DNA caused impairment in spatial memory, microglia activation and acute axonal damage (Tauber et al., 2009). Furthermore, intrathecal injection of CpG oligodeoxynucleotide (ODN) induced loss of neurons, axonal injury in the cerebral cortex, and pronounced microglia activation (Rosenberger et al., 2014). TLR9-deficient mice demonstrated reduction of inflammation, demyelination and axonal damage during the late phase of an MS mouse model (Prinz et al., 2006). It was also hypothesized that a reduced glucocorticoid receptor activity in PD due to chronically high cortisol levels could lead to TLR9 activation by components released by dopaminergic neurons, which could, in turn, exacerbate the death of these cells (Maatouk et al., 2018).

On the other hand, a neuroprotective role of microglia through activation of TLR9 has been suggested. In an AD mouse model, activation of TLR9 by CpG ODN appears to be involved in the enhanced Aβ ingestion by microglia, which reduces Aβ aggregation and improves memory impairment (Iribarren et al., 2005; Tahara et al., 2006; Doi et al., 2009; Scholtzova et al., 2009). In a mouse model of epilepsy, activation of TLR9 in microglia by DNA from degenerating neurons induced TNF-α expression. Moreover, this TLR9 activation in microglia resulted in the attenuation of aberrant neurogenesis in the hippocampus and reduced seizure mediated by kainic acid (Matsuda et al., 2015). These studies suggest that microglial TLR9 could be involved in neuroprotection in various neurodegenerative diseases.

## Conclusion and Future Perspectives

TLRs are involved in a plethora of physiological and pathological mechanisms. Activation of both endosomal and plasma membrane receptors control the microglial activity and may alter its phenotypes, which could control the evolution of neurodegenerative processes (Table 1). Thus, TLRs could represent potential pharmacological targets for the development of neuroprotective drugs. However, the current knowledge on the effects and pathways modulated by them in microglia is still modest and further studies are necessary to establish their exact roles neuropathological events. Finally, studies that specifically delete these receptors in microglia using models of neurodegeneration could contribute to clarify their roles in these pathological conditions.

**Table 1:** Expression and potential roles of TLR in neurodegenerative conditions.

TLR	Expression in diseases	Potential role		References
		Protective	Deleterious	
	↑AD, PD and ALS patients	N.A.	N.A.	Letiembre et al. (2009), Casula et al. (2011) and Doorn et al. (2014)
	APP (AD mouse model)	TLR2 deficiency impaired Aβ clearance	N.A.	Drouin-Ouellet and Cicchetti (2012)
TLR2	↑APP23 (AD mouse model)	N.A.	Increased expression associated with Aβ-plaques deposition.	Frank et al. (2009)
	↑in microglia (neuron-released α-synuclein)	N.A.	Induced neurodegeneration.	Kim et al. (2013)
	↑LPS-stimulated SOD1 G37R (ALS mouse model)	N.A.	Degeneration of motor neurons and motor axons.	Nguyen et al. (2004)
TLR3	↑MS patients	N.A.	N.A.	Bsibsi et al. (2002)
TLR4	↑AD, PD and ALS patients	N.A	N.A	Letiembre et al. (2009), Casula et al. (2011) and Drouin-Ouellet et al. (2015)
	↑APP (AD mouse model)	Boosted Aβ clearance and preserved cognition	N.A.	Herber et al. (2006), Song et al. (2011) and Sarlus and Heneka (2017)
	↑APP (AD mouse model)	N.A.	Increased expression associated with Aβ-plaques deposition.	Walter et al. (2007)
	SOD1 G93A (ALS mouse model)	N.A.	TLR4 deficiency improved motor performance and extended life expectancy.	Lee et al. (2015)
	↑AD, OPCA and PID patients	N.A.	N.A.	Bsibsi et al. (2002)
	↑MSA patients	N.A.	N.A.	Brudek et al. (2013)
TLR5	↑genetic PD mouse model	N.A.	N.A.	Letiembre et al. (2009)
	↓PD rat model	N.A.	Cognitive and motivational behavior impairment.	Viana et al. (2017)
TLR7	↑AD, PD, OPCA and PID patients	N.A.	N.A.	Bsibsi et al. (2002)
	↑genetic mice models of AD, ALS	N.A.	N.A.	Letiembre et al. (2009)
	↑genetic mice models of AD	N.A.	Deletion of TLR7 improved spatial learning.	Liu et al. (2017)
	↑TLR7 (loxoribine) in co-culture of microglia and neurons	N.A.	Loss of neurons, axonal injury in the cerebral cortex and pronounced microglia activation in the mouse brain.	Rosenberger et al. (2014)
	↑TLR7 (microRNA let-7) in mouse	N.A.	Induced neurodegeneration.	Lehmann et al. (2012a)
	↑TLR7 (ssRNA40) in mouse	N.A.	Induced neuronal cell death.	Lehmann et al. (2012b)
TLR9	↑PD patients, mouse PD and ALS models	N.A.	N.A.	Letiembre et al. (2009) and Ros-Bernal et al. (2011)
	↑TLR9 (CpG-DNA) in co-culture of microglia and neurons	N.A.	Neuronal toxicity.	Iliev et al. (2004)
	↑TLR9 (CpG-DNA) in mouse	N.A.	Impairment in spatial memory, microglial activation and acute axonal damage.	Tauber et al. (2009)
	↑TLR9 (CpG-DNA) in mouse	N.A.	Loss of neurons, axonal injury in the cerebral cortex and pronounced microglia activation.	Rosenberger et al. (2014)
	↑Late phase of MS mouse model	N.A.	Deletion of TLR9 reduced inflammation, demyelination and axonal damage.	Prinz et al. (2006)
	↑TLR9 in a PD mouse model	N.A.	Induced dopaminergic neurons loss.	Maatouk et al. (2018)
	↑TLR9 (CpG-DNA) in AD model	Aβ ingestion by microglia, which reduces Aβ aggregation and improve the memory impairment in mice	N.A.	Iribarren et al. (2005), Tahara et al. (2006), Doi et al. (2009) and Scholtzova et al. (2009)
	↑TLR9 (DNA degenerating neurons) in mouse model of epilepsy	Attenuation of aberrant neurogenesis in the hippocampus and reduced seizure	TNF-α expression	Matsuda et al. (2015)

*↑increase; ↓decrease; N.A. - not available*.

## Author Contributions

AO reviewed the literature, designed, wrote part of and revised the manuscript. CB, SS and NY reviewed the literature, wrote part of the manuscript. BF reviewed the literature, designed the manuscript and revised it.

## Conflict of Interest Statement

The authors declare that the research was conducted in the absence of any commercial or financial relationships that could be construed as a potential conflict of interest.
